# The bifunctional autophagic flux by 2-deoxyglucose to control survival or growth of prostate cancer cells

**DOI:** 10.1186/s12885-015-1640-z

**Published:** 2015-09-07

**Authors:** Jeong Yong Jeon, Seung Won Kim, Ki Cheong Park, Mijin Yun

**Affiliations:** 1 Department of Nuclear Medicine, Severance Hospital, Yonsei University College of Medicine, 134 Shinchon-dong, Seodaemun-gu, Seoul, 120-752 South Korea; 2 Brain Korea 21 PLUS Project for Medical Science, Yonsei University College of Medicine, 134 Shinchon-dong, Seodaemun-gu, Seoul, 120-752 South Korea; 3 Severance Biomedical Science Institute, Yonsei University College of Medicine, 134 Shinchon-dong, Seodaemun-gu, Seoul, 120-752 South Korea; 4 Department of Surgery, Yonsei University College of Medicine, 134 Shinchon-dong, Seodaemun-gu, Seoul, 120-752 South Korea

## Abstract

**Background:**

Recent reports using metabolism regulating drugs showed that nutrient deprivation was an efficient tool to suppress cancer progression. In addition, autophagy control is emerging to prevent cancer cell survival. Autophagy breaks down the unnecessary cytoplasmic components into anabolic units and energy sources, which are the most important sources for making the ATP that maintains homeostasis in cancer cell growth and survival. Therefore, the glucose analog 2-deoxyglucose (2DG) has been used as an anticancer reagent due to its inhibition of glycolysis.

**Methods:**

Prostate cancer cells (PC3) were treated with 2DG for 6 h or 48 h to analyze the changing of cell cycle and autophagic flux. Rapamycin and LC3B overexpressing vectors were administered to PC3 cells for autophagy induction and chloroquine and shBeclin1 plasmid were used to inhibit autophagy in PC3 cells to analyze PC3 cells growth and survival. The samples for western blotting were prepared in each culture condition to confirm the expression level of autophagy related and regulating proteins.

**Results:**

We demonstrated that 2DG inhibits PC3 cells growth and had discriminating effects on autophagy regulation based on the different time period of 2DG treatment to control cell survival. Short-term treatment of 2DG induced autophagic flux, which increased microtubule associated protein 1 light chain 3B (LC3B) conversion rates and reduced p62 levels. However, 2DG induced autophagic flux is remarkably reduced over an extended time period of 2DG treatment for 48 h despite autophagy inducing internal signaling being maintained. The relationship between cell growth and autophagy was proved. Increased autophagic flux by rapamycin or LC3B overexpression powerfully reduced cell growth, while autophagy inhibition with shBeclin1 plasmid or chloroquine had no significant effect on regulating cell growth.

**Conclusion:**

Given these results, maintaining increased autophagic flux was more effective at inhibiting cancer cell progression than inhibition of autophagic flux, which is necessary for the survival of PC3 cells. Autophagic flux should be tightly regulated to maintain metabolic homeostasis for cancer cell growth and survival in PC3 cells and is a suitable target for cancer therapy.

**Electronic supplementary material:**

The online version of this article (doi:10.1186/s12885-015-1640-z) contains supplementary material, which is available to authorized users.

## Background

Advances in surgery, hormone therapy, and chemotherapy have improved advanced prostate cancer treatments. however, these approaches are limited due to prostate cancer therapy resistance. Thus, there is a critical need to develop treatments against new cellular targets. Recently, regulation of metabolism in cancer therapy is emerging, because rapidly growing cells need plenty of the energy, nutrients, and building blocks that are required to maintain cell survival and proliferation [1–3]. Aggressive cancers consume abundant glucose to produce ATP using glycolysis, to promote the pentose phosphate pathway (PPP) to decrease oxidative stress, and to make many kinds of biomaterials [4–7]. One promising metabolic-control is chemotherapy using 2-deoxyglucose (2DG), which is a well-known glycolysis inhibitor [8, 9]. 2DG inhibits hexokinase, the rate-limiting enzyme of glycolysis, leading to depleted ATP, antioxidants, and glycolysis intermediates needed for cell survival and maintenance, thereby causing cell growth arrest and death [10–12]. Coincidently, autophagy induction rises in response to intracellular starved conditions and ER stress by 2DG as a cell survival process [3, 13].

Autophagy has an important role in the catabolic pathways that support intracellular energy sources and building blocks and clears cytotoxins to sustain homeostasis by degrading unfolded or aggregated protein and damaged cytoplasmic components with lysosomal proteases [14]. In cancer, functioning autophagy is crucial to survival and growth because rapidly proliferating cancer cells need vast energy and biomass to make new proteins, lipids, and intracellular components, and must remove protein aggregates, abnormal cytoplasmic compartments, excess reactive oxygen species, and lipid droplets to maintain the homeostasis that is produced during the development of cancer [15, 16]. These helpful functions of autophagy produce pro-survival effects in cancer development and increase resistance to chemotherapy [17, 18]. Therefore, recent reports tried to administer combination chemotherapy of both an anticancer drug and an autophagy inhibitor to block the pro-survival function of autophagy and showed a synergistic anticancer effect [19–22]. However, some groups demonstrated that autophagy contributed to the pro-death function rather than the pro-survival role. Excessive autophagy activation leads to cell death and depends on the cell types and culture environments. It is termed “autophagic cell death,” and arises from unlimited degradation of cytoplasmic components [23–26]. The double-edged sword effects of autophagy on cell survival or death are controversial [27, 28].

To determine whether autophagy is harmful or helpful for PC3 cells or LNcaP cells survival and growth under nutrient depleted conditions by 2DG, we investigated 2DG’s effect on autophagy regulation in PC3 cells and LNcaP cells, and proved that 2DG significantly suppressed both cells’ growth and promoted intense autophagic flux. Autophagic flux was differentially regulated depending on the exposure time of 2DG. Especially, increased autophagic flux significantly suppressed PC3 cells and LNcaP cells growth, and it would be blocked for cell survival.

## Methods

### Cell culture

Human prostate cancer cell line PC3 and human embryonic kidney cell line 293 T were purchased from American Type Culture Collection (ATCC, Manassas, VA) and maintained in Dulbecco’s modified Eagle medium (Gibco, Grand Island, NY, USA) with penicillin-streptomycin (100 U/mL; Gibco) and 10 % fetal bovine serum (Gibco) in a humidified atmosphere of 95 % air and 5 % CO_2_ at 37 °C.

### Cell growth assay

PC3 cells were seeded into 96-well plates (5 × 10^3^ cells per well) and incubated overnight. The next day the culture medium in each well was changed with a different concentration of 2DG containing culture medium and cultured for 3 days. After 2DG treatment, the PC3 cells on the culture plate were counted or we performed an MTT assay. Briefly, PC3 cells on the culture plate were treated with 0.5 mg/mL Thiazolyl blue tetrazolium bromide (MTT, Sigma, Louis, MO, USA) for 4 h and washed with phosphate-buffered saline (PBS). Afterwards, the plates were drained upside down on a paper towel and then solubilized with DMSO. Each well was measured using the optical density at 570 nm.

### Confocal microscopy imaging

PC3 cells were seeded on glass-bottomed cell culture dishes (NEST Biotechnology, Shanghai, China) and incubated for 24 h. The culture medium was then changed to 2DG containing culture medium and incubated for 48 h. Cells were fixed with 10 % paraformaldehyde for 30 min and washed three times with PBS. To increase permeabilization, cells were incubated in 0.25 % Triton X-100-containing PBS for 10 min. After washing the cells with a PBS blocking solution (3 % bovine serum albumin, 0.25 % Triton X-100 in PBS), cells were treated for 30 min followed by incubation overnight at 4 °C with diluted primary antibody against microtubule-associated protein 1 light chain-3B (LC3B; 1:200, Cell Signaling Technology, Danvers, MA, USA) or p62 (1:200, Santa Cruz Biotechnology, Dallas, TX, USA) in blocking solution. The next day, the cells were washed three times with PBS then incubated with diluted secondary antibody (1:500, Invitrogen, Oregon, USA) in blocking solution for 1 h. After washing the cells in PBS, Hoechst 33258 was used to stain the nucleus. The cells were analyzed using a Carl Zeiss LMS710 confocal microscope (Carl Zeiss, Gottingen, Germany).

### Fluorescence-activated cell sorting (FACS) analysis

Chemically pre-treated PC3 cells were trypsinized and harvested. Cells were fixed with 70 % ethanol for 1 h on ice and collected for permeabilization of the cell with 0.25 % Triton X-100. After centrifugation to collect the cells, we removed the supernatant and resuspended the cells with 10 μg/mL RNase and 20 μg/mL propidium iodide-containing PBS for 30 min at room temperature. The DNA content of the cells was measured using an LSRII flow cytometer (BD Bioscience, CA, USA).

### Overexpression of LC3B

PC3 cells were seeded into 6-well plates (1 × 10^4^ cells per well) and incubated overnight. LC3B or control gene plasmids were purchased from Addgene (MA, USA). Two mg of each plasmid was transfected into cultured PC3 cells using 0.2 μL/well Lipofectamine2000 (Invitrogen) according to the manufacturer’s protocols. Then LC3B-overexpressing cells were selected in culture medium containing 200 μg/mL Genectin (Invitrogen).

### Knockdown of autophagy related genes

293 T cells were seeded into 60 mm culture dishes (1 × 10^5^ cells) and incubated overnight. Lenti-viral plasmid of Beclin1 or non-targeting shRNA plasmids (1–4 μg; Sigma) were transfected into cultured 293 T cells using 20 μL/well Lipofectamine2000 according to the manufacturer’s protocols to produce virus particles. After 2 days, the culture medium of plasmid transfected 293 T cells was collected and transferred to overnight cultured PC3 cells (1 × 10^5^ cells per 100 mm dish) for virus infection. We removed the viral particle containing medium and replaced it with normal culture medium followed by cell incubation for 2 days. Afterwards, Beclin1 or non-targeting shRNA plasmid infected cells were selected in puromycin containing culture medium (2 μg/mL, Invitrogen).

### Western blot analysis

Cells were washed with PBS and lysed with 1 % sodium dodecyl sulfate (SDS) lysis buffer (60 mM Tris–HCl, pH 6.8, 1 % SDS) with protease inhibitor cocktail (Roche, Mannheim, Germany). Equal amounts of total protein from each sample were separated by SDS-polyacrylamide gel electrophoresis on 8 % or 12 % gels, and transferred onto polyvinylidene difluoride membranes (Millipore, Billerica MA, USA). Following incubation with primary antibodies against mammalian target of rapamycin (mTOR; 2972), phospho-mTOR (2448), protein kinase B (AKT; 9272), phospho-AKT (9271), AMP-activated protein kinase (AMPK; 2603), phospho-AMPK (2535), cell cycle-regulation antibody (9932, 9870; Cell Signaling Technology, Danvers, MA), p62 (SC28359; Santa Cruz Biotechnology, Dallas, Texas, USA), LC3B (L7543), and actin (A1978; Sigma), membranes were incubated with goat anti-rabbit (sc2004) or anti-mouse (sc2005) IgG horseradish peroxidase (Santa Cruz Biotechnology) as the secondary antibody. Labeled, specific protein bands were visualized using the ECL Kit (Thermo Scientific, Rockford, IL, USA).

### Chemicals

CQ (Sigma), a late-phase inhibitor of autophagy, was used at a final concentration of 20 μM for 2 h. Rapamycin (Sigma), blocker of the mTOR signaling pathway, was used as activator of autophagy at various concentrations.

### Statistical analysis

Each experiment was carried out in triplicate, and quantitative data were expressed as the mean ± S.D. Statistical analysis was conducted using the GraphPad Prism Software (San Diego, Calif., USA).

## Result

### 2DG regulates cell growth and autophagy

**Fig. 1:**
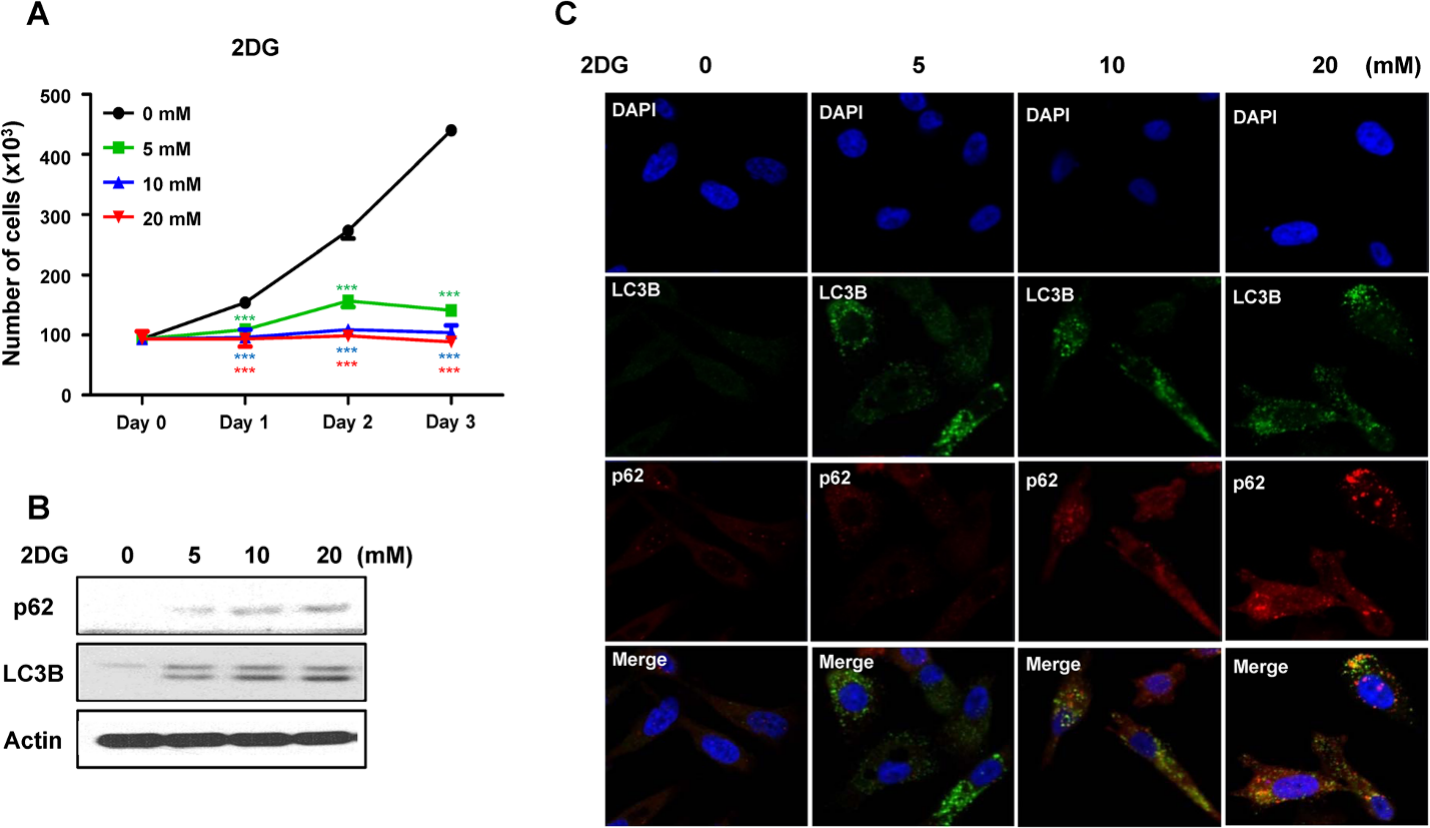
2-deoxyglucose inhibits PC3 prostate cancer cell growth and inhibits autophagy. PC3 cells were incubated with different concentrations of 2-deoxyglucose (2DG)**. a** Cell growth rate was measured by counting the cell numbers at the indicated time points for 3 days. Data are represented as means ± SD. ***P < 0.001, control versus 5 mM 2DG; ***P < 0.001, control versus 10 mM 2DG; ***P < 0.001, control versus 20 mM 2DG. (**b**) After 2DG treatment for 48 h, autophagy levels were detected using western blotting analysis. p62 was used as an autophagy substrate and LC3B showed autophagy levels. (**c**) The level of autophagy was confirmed with confocal microscopy. Blue, nucleus; Green, LC3B; Red, p62

The glycolysis inhibitor 2DG induces intracellular energy deficiency, which causes cell growth suppression and induction of autophagy. We investigated whether 2DG has these effects on PC3 prostate cancer cells. The growth of PC3 cells was significantly inhibited by 2DG in a dose dependent manner, and the suppression effects of 2DG were strongly observed even at a 5 mM 2DG concentration (Figure 1a). Autophagy was inhibited by 2DG. This was confirmed by LC3B expression levels and highly accumulated autophagy substrate p62 levels in the western blots (Fig. 1b) and confocal imaging (Fig. 1c). As shown in Fig. 1b and c, the level of accumulated p62 gradually increased with higher 2DG concentration. These effects of 2DG were similarly observed in LNCaP prostate cancer cells (Additional file 1: Figure S1A and S1B). 2DG inhibits cell growth and autophagy.

### 2DG controls the cell cycle of PC3 cells

**Fig. 2:**
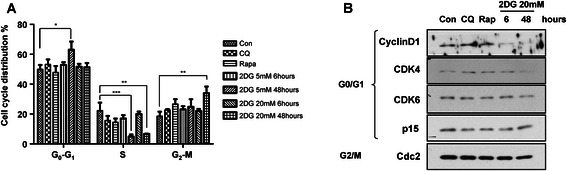
Long-term exposure to 2DG enhances cell cycle arrest. PC3 cells were treated with 2DG for a short time (6 h) or long time (48 h). CQ was used as an autophagy inhibitor at a final concentration of 20 μM for 2 h. Rapamycin was used as an autophagy inducer at 100 nM for 6 h. **a** After 2DG treatment, each cell was collected for FACS analysis. Cells were stained with iodine for 5 min and analyzed for the amount of DNA content. Each DNA content rate is expressed as G1, S, and G2 phase. Data are represented as the means ± SD. *P < 0.05, **P < 0.01, **P < 0.001. (**b**) Expression levels of cell cycle related genes were observed using western blotting analysis

We wondered how 2DG controls the growth of PC3 cells. We observed the changes in cell cycle based on 2DG concentration and treatment time. There were no significant changes in cell cycle with a short-term treatment of 2DG. However, long-term treatment of 2DG strongly increased the G0/G1 phase and decreased the S phase in 5 mM 2DG treated PC3 cells. Interestingly, higher concentrations of 2DG remarkably decreased the S phase and increased the G2 phase rather than changing the G0/G1 phase (Fig. 2a). To check the relationship between the cell cycle and autophagy, we administered chemical regulators of autophagy using the autophagy inhibitor CQ or the autophagy inducer rapamycin for 6 h in PC3 cells. Both regulators showed no statistically significant effects on cell cycle arrest, and only rapamycin increased G2 phase arrest. These results were confirmed with western blot. The expression level of G0/G1 or G2/M related markers, cyclinD1, cyclin dependent kinase 4 (CDK4), and cell division control protein 2 (cdc2) was decreased, but CDK6 and cyclin dependent kinase inhibitor 2b (p15) were not changed by 2DG, which was remarkably shown with the 2DG treatment condition for 48 h (Fig. 2b). LNCaP cells also showed cell cycle arrest that the expression level of cyclinD1, p15 and cdc2 were significantly decreased but the expression level of CDK4 was not changed (Additional file 1: Figure S1C). Taken together, 2DG induced G0/G1 arrest at the early phase and G2/M arrest at the late phase of autophagy induction, inducing cell cycle arrest.

### Autophagy is differentially regulated by 2DG

**Fig. 3:**
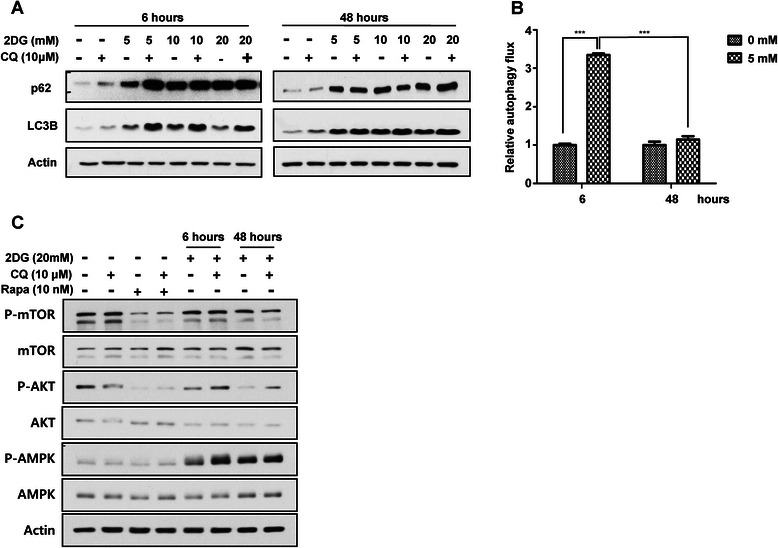
Autophagic flux is differentially regulated by exposure time to 2DG. 2DG was administered to PC3 cell, with CQ or alone, to confirm autophagic flux. **a** Protein levels of p62 and LC3B in 2DG containing culture medium for the indicated time followed by CQ or no further treatment were observed with western blotting. L.E. means long exposure. (**b**) Autophagic flux calculated by the accumulated amount of LC3B with treatment of CQ autophagy blocker for 2 h. Data are represented as the means ± SD. ***P < 0.001 for both culture conditions. (**c**) The changes in intracellular signaling pathway related autophagy regulation were verified for each condition. Phosphorylated levels of mTOR and AKT were checked for whether the inhibitory signal of autophagy was induced. The AMPK signaling pathway was confirmed as an autophagy activating condition

We observed that 2DG significant inhibited PC3 cell growth and blocked autophagy, but cell cycle arrest was increased by autophagy induction. Because of these interesting results, we checked the relationship between autophagy regulation and 2DG. We measured the autophagic flux with a lysosomal protease inhibitor, CQ, which blocks proteolysis in the lysosome and leads to the accumulation of LC3B and p62. We analyzed the amount of accumulated LC3B and p62, which indicates autophagic flux. Western blotting showed that the accumulated LC3B and p62 levels with CQ treatment for 2 h were strongly increased by short-term treatment with 2DG for 6 h compared to the control treatment condition and were enhanced further with higher concentrations. However, there were no differences seen with long-term treatment of 2DG for 48 h, either with CQ or alone. CQ did not affect p62 and LC3B accumulation (Fig. 3a). As shown in Fig. 3b, Additional file 2: Figure S2A and S2B, 2DG noticeably increased autophagic flux at the early phase, but significantly inhibited autophagic flux in PC3 cells and LNCaP cells.

Next, we observed changes in intracellular signal pathways related to autophagy regulation. In autophagy regulation, mTOR and AMPK are representative signaling pathways. mTOR has a negative role by inhibiting unc-51-like autophagy activating kinase 1 (ULK1) phosphorylation, which prevents autophagy initiation. mTOR is inhibited by the autophagy inducing chemical rapamycin. In contrast, AMPK has positive role by inhibiting the mTOR pathway [29]. Western blotting results showed that the phosphorylated level of mTOR/AKT was decreased in a time-dependent manner, but phosphorylated level of AMPK was strongly increased by treatment with 2DG. The phosphorylated level of AMPK was increased in LNCaP cells by 2DG. These changes of intracellular signaling pathways were positive for autophagy induction and continued for 48 h of 2DG treatment (Fig. 3c and Additional file 2: Figure S2C).

These results mean that autophagic flux was differentially regulated over the time period of 2DG treatment. Increased autophagic flux in the early phase of 2DG treatment was eventually inhibited by the expanded time of 2DG treatment, although the autophagy inducing signal was constantly transduced.

### Autophagic flux regulates cell growth and survival

**Fig. 4:**
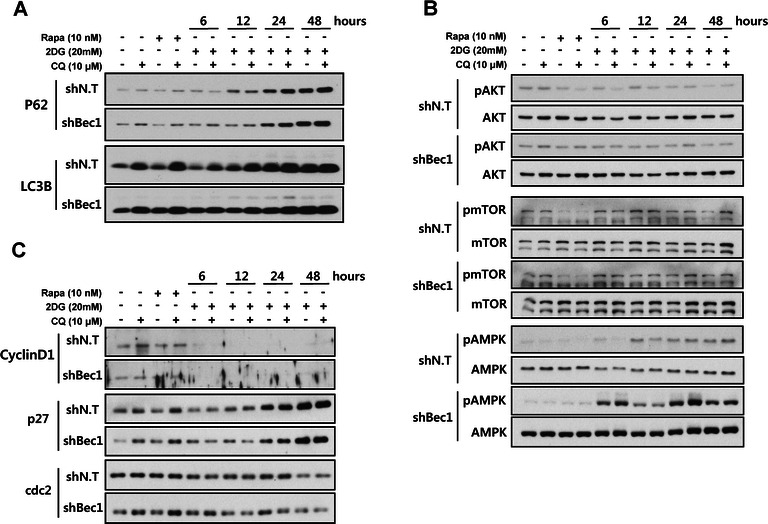
Knockdown of Beclin1 is not effective at regulating the cell cycle. The samples for western blotting were prepared in each culture condition. Afterwards, **a** changing autophagy levels, (**b**) the kinetics of signaling proteins in autophagy regulation, and (**c**) the expression levels of cell cycle related genes were checked in both normal and Beclin1 knocked-down PC3 cells using shRNA introduction. shN.T., non-targeting shRNA; shBec1, shRNA for Beclin1

We investigated whether autophagy regulates the cell cycle or survival of PC3 cells. To confirm the relationship between autophagy inhibition and cell growth, we knocked-down Beclin1, an important component of the autophagy initiation process. PC3 cells were transfected with a shBeclin1 or shNon-target (shNT) plasmid. Beclin1 knocked-down PC3 cells showed accumulated p62 levels in normal conditions, but there were no significant differences between only Beclin1 knocked-down culture conditions and 2DG added conditions (Fig. 4a). In the autophagy-regulating signaling pathways, phosphorylated levels of mTOR/AKT between shBeclin1 and shNT transduced PC3 cells under each condition were not different. Only the AMPK signaling pathway was intensely activated by the autophagy knockdown in 2DG treated conditions compared with normal culture conditions (Fig. 4b). This means that autophagy knockdown was not critical for intracellular signal transduction.

**Fig. 5:**
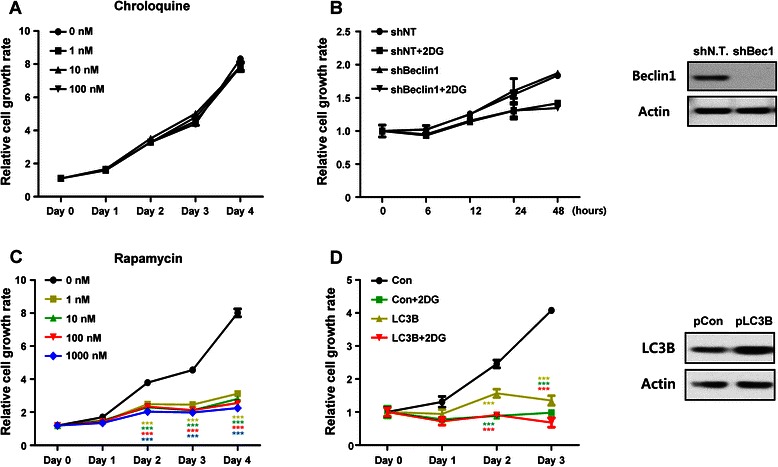
Induction of autophagy suppresses cell growth and survival. Autophagic flux was regulated by chemical treatment or genetic modification to prove the relationship between autophagic flux and cell growth. Autophagy was blocked with **a** various concentrations of CQ treatment or (**b**) Beclin1-targeting shRNA. The level of Beclin1 expression was confirmed with western blotting for Beclin1 (Right panel). (**c**) Autophagy was induced with rapamycin at the indicated concentrations. The level of LC3B expression was confirmed with western blotting for LC3B (Right panel). Data are represented as the means ± SD, ***P < 0.001 versus 1 nM, ***P < 0.001 versus 10 nM, ***P < 0.001 versus 100 nM, ***P < 0.001 versus 1000 nM. (**d**) The LC3B plasmid to overexpress LC3B was introduced into PC3 cells. Data are represented as the means ± SD. ***P < 0.001 versus 2DG, ***P < 0.001 versus LC3B, ***P < 0.001 versus LC3B + 2DG. Cell growth rate was measured using an MTT assay (**a** and **c**) or cell counting (**b** and **d**). Autophagy blocking did not show a synergistic effect with 2DG to regulate cell growth. However, autophagy induction suppressed cell growth, not only with LC3B overexpression, but also with 2DG combination treatment, which showed a synergistic cell suppression effect

In addition, expression levels of cell cycle regulated genes were not different in shBeclin1 plasmid transduced PC3 cells than in shNT plasmid bearing PC3 cells under any condition (Fig. 4c). We investigated whether autophagy inhibition can suppress PC3 cell growth. PC3 cells were blocked for autophagic flux using the chemical inhibitor CQ (Fig. 5a) or genetic modification with shRNA plasmids to check cell growth (Fig. 5b). The level of Beclin1 expression was confirmed with western blot and undetectable in shBeclin1 plasmid bearing PC3 cells (Fig. 5b right panel). Under both conditions, PC3 cell growth was not different between normal conditions and 2DG treated conditions (Fig. 5a and b). LNCaP cells did not show any growth inhibiting effect by autophagy inhibition (Additional file 3: Figure S3A). Although the CQ concentration was increased, CQ had no effect on the cell growth of both cells (Fig. 5a and Additional file 3: Figure S3A). After observing no relationship between autophagy blocking and cell growth, we established an LC3B overexpressing PC3 cell line to confirm that autophagy activation has an inhibitory effect on cell growth. The level of LC3B expression was confirmed with western blot and was strongly increased in LC3B plasmid bearing PC3 cells. Chemical activation of autophagy with rapamycin strongly inhibited the growth of PC3 and LNCaP cells in a dose dependent manner and showed a noticeable effect even at very low concentrations of 1 nM (Fig. 5c and Additional file 3: Figure S3B). Autophagy activation by LC3B overexpression very intensely inhibited PC3 cell growth similar to 2DG treated conditions. Interestingly, we observed that the survival of LC3B overexpressing PC3 cells decreased up to 20 % with 2DG treatment (Fig. 5d). These results indicate that cell growth and cell survival are related to autophagy activation rather than autophagy inhibition.

## Discussion

This study was carried out with the hypothesis that 2DG controls cell growth and survival through autophagy regulation. 2DG triggers intracellular energy deficiency via glycolysis inhibition and increased endoplasmic reticulum (ER) stress, which induces cell death and arrest [9, 30]. Recent studies reported that genetic or chemical cancer cell metabolism manipulation, including 2DG treatment, showed remarkable antitumor effects [31, 32]. Cheong et al. demonstrated that 2DG induced cell death and showed synergistic effects using metformin co-treatment conditions [33]. Our data proved the anticancer effects of 2DG in prostate cancer cells by inhibiting cell proliferation, but did not show strong cell death (Fig. 1). These results were somehow different with the Cheong group study. However, these antitumor effects were dependent on the different types of cancer cells confirmed by other groups. Various types of malignant cancers showed only a slowdown of cell growth rather than cell death, and the administration of only 2DG had no significant effects *in vivo* [8, 34]. However, it is clear that 2DG has a strong inhibition effect on cancer cell growth.

2DG promotes these effects through decreased intracellular ATP levels and ER stress by blocking glycolysis, which leads to autophagy induction. In this study, we found that increased autophagy with rapamycin or LC3B overexpression strongly suppressed PC3 cell growth (Fig. 5c and d). 2DG also increased autophagic flux at the early phase and suppressed the growth of PC3 and LNCaP cells (Fig. 1a and Additional file 1: Figure S1A). Overall, 2DG induced PC3 cell growth inhibition arose by autophagy activation rather than autophagy inhibition.

However, we wondered why increased autophagic flux at the early phase of 2DG treatment was gradually decreased at 48 h of treatment with 2DG although the AMPK signal, an autophagy inducing internal signaling pathway, were constantly activated (Fig. 3).

Our results showed that the decreased mTOR/AKT signaling pathway and increased AMPK signaling pathway were continuously maintained by 2DG until the last time points of sample preparation (Fig. 3b). AMPK is well known as an intracellular energy sensor that has the ability to detect the AMP:ATP ratio. In low levels of ATP due to exercise or metabolic stress, AMPK is activated to inhibit energy consuming anabolic pathways and promote energy producing catabolic pathways like glucose uptake, glycolysis, and beta oxidation to increase ATP levels. In addition, AMPK tightly regulates autophagy through the inhibition of the mTOR signaling pathway [35, 36].

One possibility for autophagy blocking by 2DG with long-term treatment is lower levels of glucose and ATP in the cytoplasm. ATP is a very important energy source to maintain metabolic homeostasis, and is used as a substrate to transfer energy for transducing signals by kinase, as a material to make nucleic acids, and it participates in biosynthetic processes [37, 38]. In addition, ATP is a very critical for the process of autophagy maturation [39]. Autophagy begins with phagophore formation with a small double membrane, and the end of the phagophore is elongated. After then it engulfs the described substrate and encloses the double membrane. This is called the autophagosome. Many autophagy related proteins including ULK2, the autophagy related (ATG) proteins, the focal adhesion kinase family interacting protein FIP200, Beclin1, the human phosphatidylinositol 3 kinase adaptor protein p150, the class III phosphatidylinositol-3 kinase VPS34, and LC3 participate in this process. The autophagosome fuses with the lysosome to construct the autolysosome that degrades the autophagosome contained substrates to recycle biomaterials [40]. ATP is required for these sequestrations by autophagosome [41, 42]. ATP has a crucial role in maintenance of V-ATPase activity that produces lysosomal acidification to optimize lysosomal hydrolases within the lysosomal membrane. V-ATPase consists of ATP hydrolysis-acting V_1_ and proton translocation-acting V_0_. Lower levels of ATP due to limited glucose supply promotes separation of these two components and causes loss of V-ATPase activity, which inhibits autophagic flux and blocks autophagy cargo degradation by lysosomal proteases [43–45]. Similarly, long-term treatment of 2DG prohibits glycolysis and sufficient ATP synthesis, which reduces autolysosome conformation and autophagic flux. This could partially explain the inhibition of autophagic flux by 2DG. Because ATP is synthesized not only by glycolysis, but also beta oxidation in the mitochondria, blocking of glycolysis with 2DG did not perfectly inhibit ATP synthesis. Supplements of glutamine via glutaminolysis and pyruvates to the mitochondria could make ATP [46]. So, in our experimental conditions, the reason for inhibition of autophagic flux by depletion of ATP with 2DG treatment is not enough.

Another possibility is a defense mechanism for survival in cells. Recent reports use the term "autophagic cell death," which means unlimited autophagy activation. It leads to cell death because autophagy uptakes and degrades not only the properly eliminated proteins or organelles, but also the normal intracellular components that work well in the cell [28]. Some groups reported the possibility of clinical cancer therapy using autophagy inducers that promote autophagic cell death in cancer cells [28, 47, 48]. In cancer cells, this autophagic cell death is thoroughly prevented for survival, so does 2DG treated PC3 cells. In our results, blocking of autophagy through chemical or genetic inhibitory manipulation did not have significant anticancer effects compared with normal culture conditions. Knockdown of an autophagy related gene showed only increased autophagic flux blocking and little change in cell cycle related gene expression (Fig. 4). In addition, inhibition of autophagic flux by CQ or shRNA showed no remarkable difference in PC3 cell growth (Fig. 5a and b). In contrast, overactivation of autophagy by both rapamycin and overexpression of LC3B significantly suppressed cancer cell growth, and additional treatment of 2DG into overactivated autophagy conditions strongly decreased PC3 cell viability (Fig. 5c and d). In LNCaP cells, the effect of cell growth inhibition by rapamycin was more intense than in PC3 cells (Additional file 3: Figure S3B).

Although cell death through autophagy was very dependent on cell type and culture environments, recent reports demonstrated that increased autophagic flux has strong anticancer effects [24, 49, 50]. Zhao et al. report that acetylated Foxo1 interacted with Atg7 and E1-like protein and the oxidative stress increased autophagic flux, leading to cell death. Similar to our results, excessive autophagy activation by increased intracellular ER stress and ROS levels induced cell death [50]. Taken together, metabolic stress causes autophagy activation and leads to cell death. However, the detailed mechanism for the regulation of autophagic flux in cancer cell survival is not clear and needs to be studied further [27, 28].

## Conclusion

2DG treatment inhibits PC3 cell growth and differentially regulates autophagic flux time dependently. Short-term treatment of 2DG strongly induces autophagic flux, which causes cell growth inhibition, but long-term treatment blocks the autophagic flux needed for PC3 cell survival and maintenance. Although these findings need more detailed molecular and physiological mechanisms for autophagy regulation, these results are important evidence for exploring new therapeutic agents for cancer by inducing autophagy and maintaining excessive autophagic flux.

## Additional files

Additional file 1: Figure S1. 2-deoxyglucose regulates LNCaP prostate cancer cell growth, autophagy and cell cycle. LNCap cells were incubated with different concentrations of 2-deoxyglucose (2DG)**.** (A) Cell growth rate was measured using MTT assay at the indicated time points for 3 days. Data are represented as means ± SD. ****P* < 0.001, control versus 5 mM 2DG; ****P* < 0.001, control versus 10 mM 2DG; ****P* < 0.001, control versus 20 mM 2DG. (B) After 2DG treatment for 48 h, autophagy levels were detected using western blotting analysis. p62 was used as an autophagy substrate and LC3B showed autophagy levels. (C) LNCaP cells were treated with 2DG for a short time (6 h) or long time (48 h). CQ was used as an autophagy inhibitor at a final concentration of 20 μM for 2 h. Rapamycin was used as an autophagy inducer at 100 nM for 6 h. Expression levels of cell cycle-related genes were observed using western blotting analysis. Long-term exposure to 2DG enhances cell cycle arrest. (TIFF 2456 kb)
Additional file 2: Figure S2. Autophagic flux is differentially regulated by exposure time to 2DG. 2DG was administered to LNCaP cells, with CQ or alone, to confirm autophagic flux. (A) Protein levels of p62 and LC3B in 2DG-containing culture medium for the indicated time followed by CQ or no further treatment were observed with western blotting. (B) Autophagic flux calculated by the accumulated amount of LC3B with treatment of CQ for 2 h. Data are represented as the means ± SD. ****P* < 0.001 for both culture conditions. (C) The changes in intracellular signaling pathway related autophagy regulation were verified for each condition. Phosphorylated level of mTOR was checked for whether the inhibitory signal of autophagy was induced. The AMPK signaling pathway was confirmed as an autophagy activating condition. (TIFF 3616 kb)
Additional file 3: Figure S3. Induction of autophagy suppresses cell growth and survival. Autophagic flux was regulated by chemical treatment to prove the relationship between autophagic flux and cell growth. (A) Autophagy was blocked with various concentrations of CQ treatment (B) Autophagy was induced with rapamycin at the indicated concentrations. Data are represented as the means ± SD, ****P* < 0.001 versus 1 nM, ****P* < 0.001 versus 10 nM, ****P* < 0.001 versus 100 nM, ****P* < 0.001 versus 1000 nM. Cell growth rate was measured using an MTT assay. Autophagy blocking did not show a synergistic effect with 2DG to regulate cell growth. However, autophagy induction significantly suppressed cell growth. (TIFF 1295 kb)

## Abbreviations

2DG: 2-Deoxyglucose
LC3B: Microtubule-associated protein 1 light chain-3B
CQ: Chloroquine
mTOR: Mammalian target of rapamycin
AMPK: AMP-activated protein kinase
