# Supplementary figures and images for: The bifunctional autophagic flux by 2-deoxyglucose to control survival or growth of prostate cancer cells
Source: BMC Cancer. 2015 Sep 7;15:623. doi: 10.1186/s12885-015-1640-z (PMC4562121; doi:10.1186/s12885-015-1640-z)

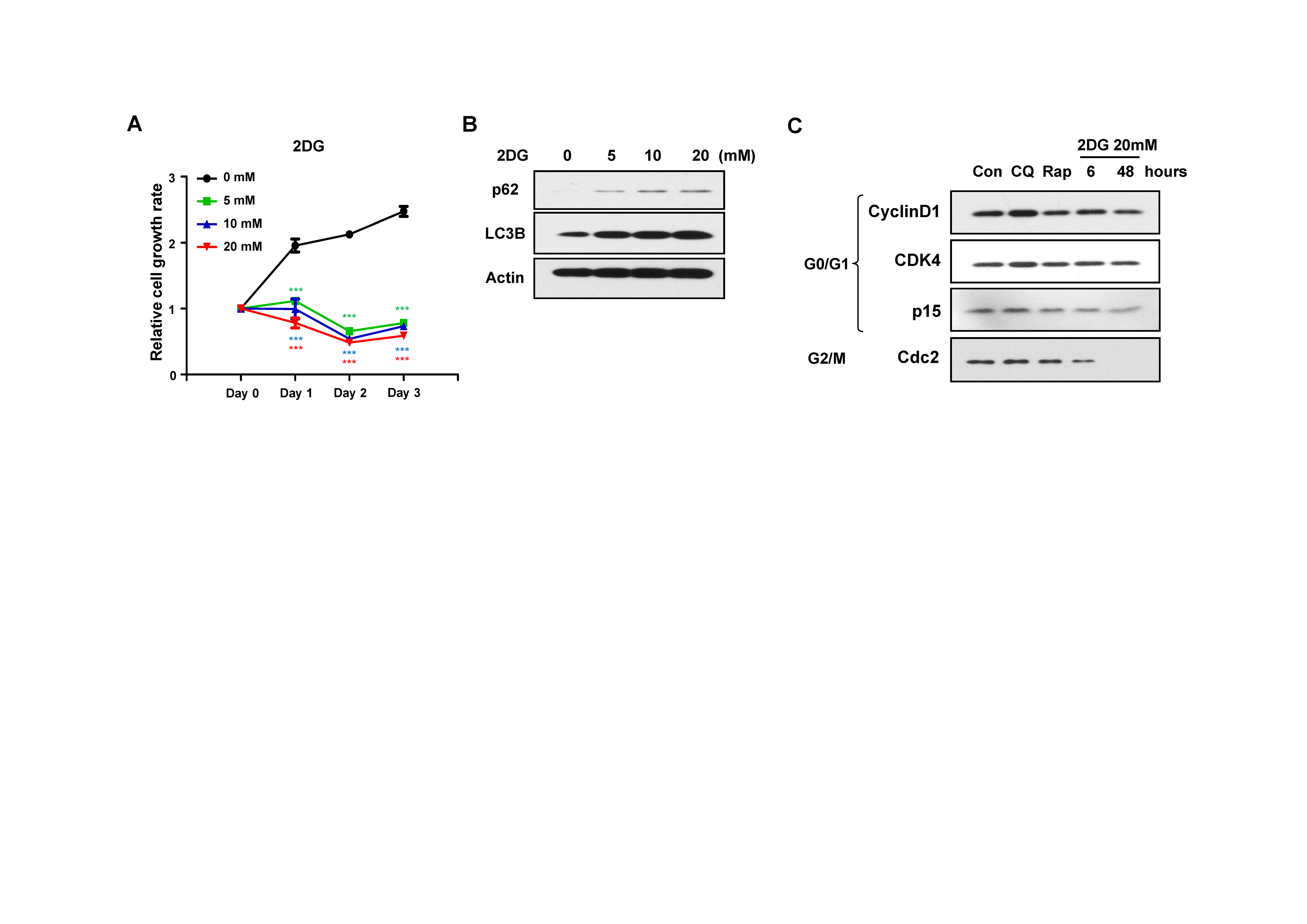

Supplement: Additional file 1: Figure S1. — 2-deoxyglucose regulates LNCaP prostate cancer cell growth, autophagy and cell cycle. LNCap cells were incubated with different concentrations of 2-deoxyglucose (2DG). (A) Cell growth rate was measured using MTT assay at the indicated time points for 3 days. Data are represented as means ± SD. ***P < 0.001, control versus 5 mM 2DG; ***P < 0.001, control versus 10 mM 2DG; ***P < 0.001, control versus 20 mM 2DG. (B) After 2DG treatment for 48 h, autophagy levels were detected using western blotting analysis. p62 was used as an autophagy substrate and LC3B showed autophagy levels. (C) LNCaP cells were treated with 2DG for a short time (6 h) or long time (48 h). CQ was used as an autophagy inhibitor at a final concentration of 20 μM for 2 h. Rapamycin was used as an autophagy inducer at 100 nM for 6 h. Expression levels of cell cycle-related genes were observed using western blotting analysis. Long-term exposure to 2DG enhances cell cycle arrest. (TIFF 2456 kb) [file 12885_2015_1640_MOESM1_ESM.tiff]

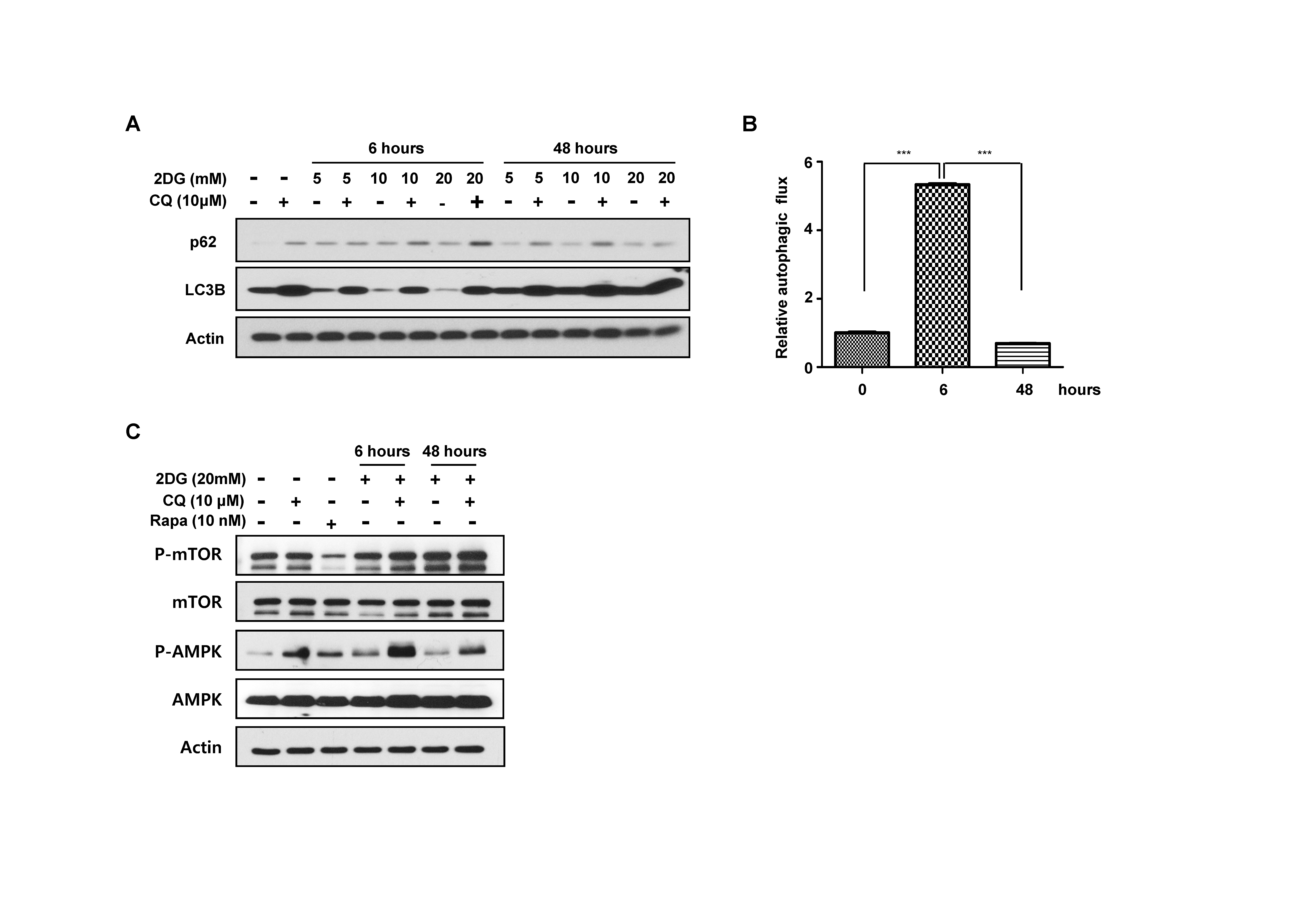

Supplement: Additional file 2: Figure S2. — Autophagic flux is differentially regulated by exposure time to 2DG. 2DG was administered to LNCaP cells, with CQ or alone, to confirm autophagic flux. (A) Protein levels of p62 and LC3B in 2DG-containing culture medium for the indicated time followed by CQ or no further treatment were observed with western blotting. (B) Autophagic flux calculated by the accumulated amount of LC3B with treatment of CQ for 2 h. Data are represented as the means ± SD. ***P < 0.001 for both culture conditions. (C) The changes in intracellular signaling pathway related autophagy regulation were verified for each condition. Phosphorylated level of mTOR was checked for whether the inhibitory signal of autophagy was induced. The AMPK signaling pathway was confirmed as an autophagy activating condition. (TIFF 3616 kb) [file 12885_2015_1640_MOESM2_ESM.tiff]

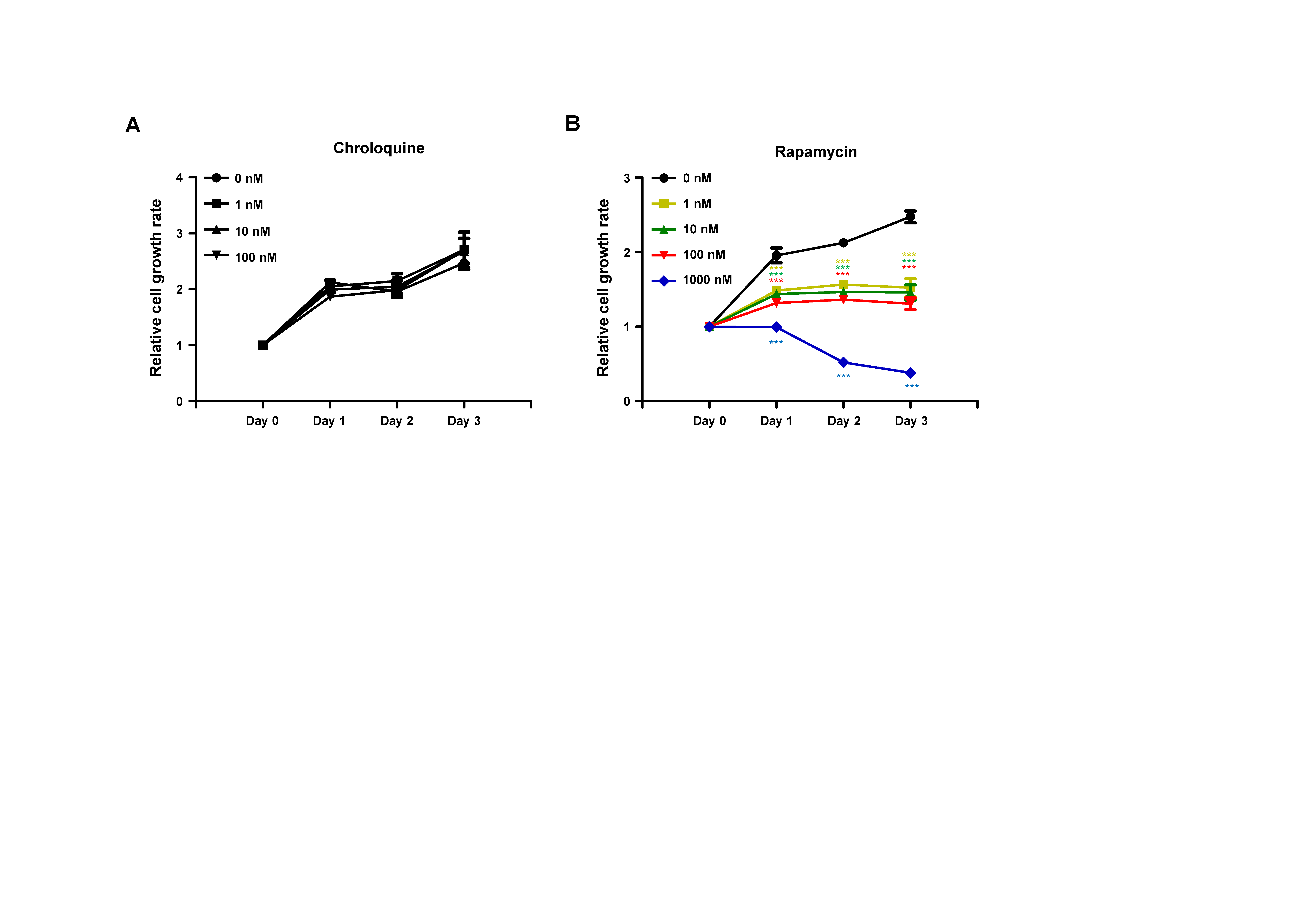

Supplement: Additional file 3: Figure S3. — Induction of autophagy suppresses cell growth and survival. Autophagic flux was regulated by chemical treatment to prove the relationship between autophagic flux and cell growth. (A) Autophagy was blocked with various concentrations of CQ treatment (B) Autophagy was induced with rapamycin at the indicated concentrations. Data are represented as the means ± SD, ***P < 0.001 versus 1 nM, ***P < 0.001 versus 10 nM, ***P < 0.001 versus 100 nM, ***P < 0.001 versus 1000 nM. Cell growth rate was measured using an MTT assay. Autophagy blocking did not show a synergistic effect with 2DG to regulate cell growth. However, autophagy induction significantly suppressed cell growth. (TIFF 1295 kb) [file 12885_2015_1640_MOESM3_ESM.tiff]
